# Technical specifications of dose management systems: An international atomic energy agency survey

**DOI:** 10.1002/acm2.14219

**Published:** 2023-12-07

**Authors:** Ioannis A. Tsalafoutas, Laurentcia Arlany, Egor Titovich, Yaroslav Pynda, Ricardo Ruggeri, Roberto Mariano Sánchez, Ingrid Reiser, Virginia Tsapaki

**Affiliations:** ^1^ Hamad Medical Corporation, Occupational Health and Safety Medical Physics Section Doha Qatar; ^2^ Sengkang General Hospital Singapore Singapore; ^3^ Dosimetry and Medical Radiation Physics Section International Atomic Energy Agency Vienna Austria; ^4^ Division of Human Health International Atomic Energy Agency Vienna Austria; ^5^ Fundación Médica de Río Negro y Neuquén‐Leben Salud Rio Negro Argentina; ^6^ Servicio de Física Médica Hospital Clínico San Carlos Madrid Spain; ^7^ Department of Radiology The University of Chicago Chicago Illinois USA

**Keywords:** dose management systems, optimization, patient radiation dose, radiological examinations

## Abstract

**Purpose:**

Dose management systems (DMS) have been introduced in radiological services to facilitate patient radiation dose management and optimization in medical imaging. The purpose of this study was to gather as much information as possible on the technical characteristics of DMS currently available, regarding features that may be considered essential for simply ensuring regulatory compliance or desirable to fully utilize the potential role of DMS in optimization of many aspects of radiological examinations.

**Methods:**

A technical survey was carried out and all DMS developers currently available (both commercial and open source) were contacted and were asked to participate. An extensive questionnaire was prepared and uploaded in the IAEA International Research Integration System (IRIS) online platform which was used for data collection process. Most of the questions (93%) required a “Yes/No” answer, to facilitate an objective analysis of the survey results. Some free text questions and comments’ slots were also included, to allow participants to give additional information and clarifications where necessary. Depending on the answer, they were considered either as “Yes” or “No.”

**Results:**

Given the way that the questions were posed, every positive response indicated that a feature was offered. Thus, the percentage of positive responses was used as a measure of adherence. The percentages of positive answers per section (and sub‐section) are presented in graphs and limitations of this type of analysis are discussed in detail.

**Conclusions:**

The results of this survey clearly exhibit that large differences exist between the various DMS developers. Consequently, potential end users of a DMS should carefully determine which of the features available are essential for their needs, prioritize desirable features, but also consider their infrastructure, the level of support required and the budget available before selecting a DMS.

## 1 INTRODUCTION

Dose management systems (DMS) were introduced in radiological services about a decade ago, to facilitate patient radiation dose and quality management, quality assurance and optimization in medical imaging.^1^, ^2^, ^3^, ^4^, ^5^, ^6^, ^7^, ^8^, ^9^ The systems are used to automatically monitor all radiological examinations, to collect and evaluate data regarding patient dose related parameters, patient demographics, and other types of data. They are considered as an essential tool for ensuring regulatory compliance, but also for improving the efficiency of quality assurance programs.^2^ As reported in the recent literature, DMS facilitate the optimization process and the establishment of best practices, among which one of the most important is to use the lowest possible radiation dose that guarantees that the image quality obtained is appropriate the respective diagnostic task.^9^, ^10^, ^11^, ^12^, ^13^, ^14^

Depending on the type and specifications of each DMS, apart from quantities relevant to patient radiation dose, other data can be collected and utilized to further enhance patient care and assist with continuous practice quality improvement, as, for example, regarding the optimum use of contrast media. DMS have different features and capabilities which often determine their fields of application, and not all DMS are applicable for all tasks. DMS come in many configurations, some being developed by commercial companies, whereas others are available for free, as an open‐source solution. Depending on their specifications, DMS provide various levels of automation, and different ways of collection, analysis, and reporting of results, but in general, they are more efficient for improving the everyday clinical practice, compared to previous manual or semi‐automated data collection methods.^5^

The International Atomic Energy Agency (IAEA), since many years ago, has acknowledged the importance of DMS in radiation protection of patients within the imaging departments. It has held technical meetings^15^, ^16^ and informative educational events on the subject,^17^ while recently provided guidance which underlined the importance of DMS in everyday clinical practice.^1^, ^2^ With the evolution of medical imaging technology, DMS has also evolved and currently there is a large variety of DMS for the users to choose from, depending on their specific needs and financial resources. However, there is little guidance on how to set up and use such a system optimally. There is also a lack of standardized procedures related to acceptance testing and periodic quality assurance tests of DMS. For all these reasons, the IAEA is developing guidance on the content, analysis, and evaluation of these systems to help Member States understand, set up, and use them appropriately. The guidance will include information on basic concepts, technology, technical features, and advanced functionalities of DMS, acceptance, commissioning and quality control testing, as well as use cases and examples. Within this context, a technical survey both for commercial and open‐source systems was developed to gather as much information as possible and analysis of the results is being presented here.

## 2 METHODS

A questionnaire with 302 questions was developed, 93% of which required either “Yes/No” answers or selection from a list of pre‐set answers. It was divided into six sections and the information collected was related to certifications (Section 1), data transfer methods (Section 2), operational parameters and dose metrics collected or calculated per supported modality (Section 3), statistical and reporting capabilities (Section 4), customization capabilities regarding examination identification, grouping and alert setting features (Section 5), and finally, implementation process and technical support information (Section 6). Each section included a number of free text questions for the following reasons: 1) to capture nuanced responses, 2) to encourage respondents to share their unique perspectives, ideas, or experiences that may not have been considered in the survey design, since by allowing respondents to freely express themselves, their answers may reveal unexpected or unanticipated insights, 3) to offer some flexibility and adaptability to answer the questions asked, and 4) to investigate the potential of a qualitative data analysis, depending on the collection of detailed technical data info that could probably allow an in depth analysis of the DMS capabilities. At the end of each section and subsection, “questions” were added (comments, remarks, file upload) to offer the possibility to the DMS developers to provide comments, clarifications or upload documentation related to the respective questions. The questionnaire was sent to all known commercial and open source DMS developers at that time (June 2022).

Data collection was done using the IAEA International Research Integration System (IRIS) online platform.^18^ IRIS is a digital platform, launched in 2020, that aimed at streamlining data collection process and enhancing quality of scientific research. It provides access to trustworthy and validated data and facilitates the collection and collation of research data, the distribution of surveys, and the management of datasets. The platform supports the collection of numerical and text data, images, as well as DICOM images with subsequent tag extraction. It is hosted on the secure IAEA cloud infrastructure, ensuring top‐level security at all stages of the data collection and management process.

Data was collected during the period from October 2022 until April 2023. The evaluation of the responses was accomplished through statistical analysis implemented in Microsoft Excel.

## 3 RESULTS

Before presenting the results of the survey, it is important to state that the IAEA's intention in sharing these data is solely to provide valuable information and insights to the scientific community and stakeholders. The publication of these findings should not be interpreted as an endorsement or promotion of any developer mentioned in the survey. The IAEA remains neutral and impartial in its role as a facilitator of knowledge exchange, ensuring that the information is disseminated objectively and without favoritism. The ultimate goal is to foster transparency, facilitate informed decision‐making, and promote the advancement of scientific knowledge in the field, while maintaining an unbiased stance towards the DMS developers involved. For this reason, the results that will be presented in the graphs have been anonymized by assigning each developer with a number.

**FIGURE 1:**
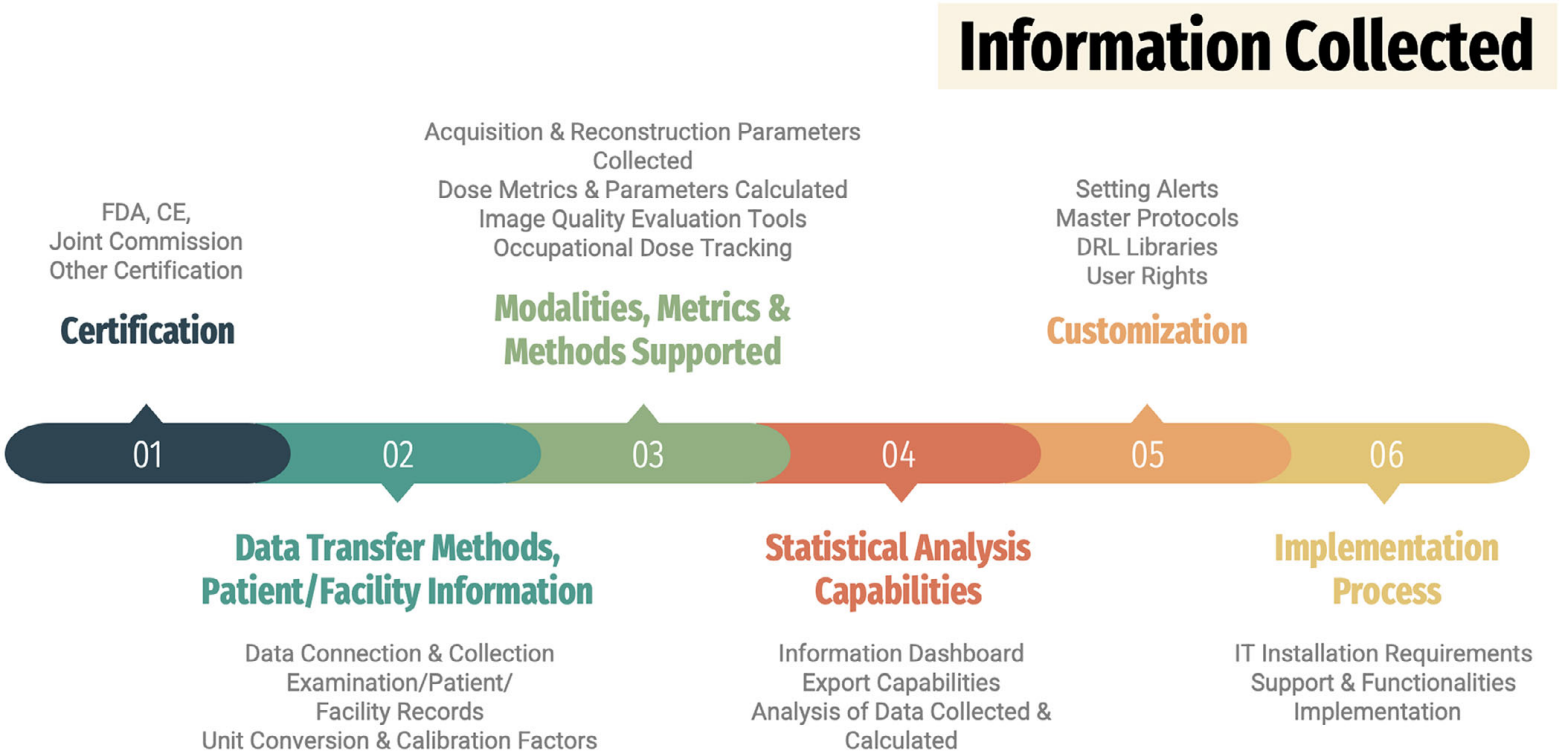
Overview of information collected from DMS developers during the survey.

Fourteen (14) DMS developers were contacted, and after briefly informed about the purpose of this survey, they were asked to participate. Twelve responded and 11 of them completed the whole or a considerable part of the questionnaire (eight commercial, two open‐source, and one in‐house systems). The DMS developers that participated in the survey were the following in an alphabetical order: Bayer, General Electric HealthCare‐GE Medical Systems, Hospital Clinico San Carlos, INFINITT Europe GmbH, Medsquare, OpenREM (Copyright the Royal Marsden), PACSHealth LLC, PixelMed Publishing LLC, Qaelum NV, Region Västerbotten, and Siemens Healthineers. This order does not correspond to the number used for each DMS developer. The data collected using IRIS was exported to Microsoft Excel for subsequent analysis. A graphical summary of the information collected during the survey, describing Sections 1−6, is presented in Figure 1.

The initial step was to identify the percentage of questions that each respondent answered positively, and questions answered negatively or were not answered at all (NA) were summed‐up together as negative. In “Yes/No” answers, this was straightforward. In the case of free‐text questions, the responses had to be reviewed one‐by‐one to decide whether the answer was positive or negative. To facilitate the reading of the results in the final table, “Yes” answers were represented with a green check mark (**✓**), “No” answers were represented with a red “X” mark (**✗**), and NA answers were represented with an orange exclamation mark (**!**). To facilitate the reading of free‐text answers, those considered as “Yes” were represented with green color letters and those considered as “No” were represented with red color letters.

**FIGURE 2:**
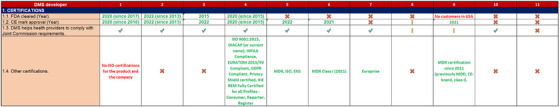
Summary of the DMS survey responses regarding Section 1 (Certification).

The results of the survey, containing the full list of questions and answers, were 17 pages long, though effort was made to summarize as much as possible the free text answers, to increase the width of the questions’ column and decrease its height, to reduce the number of pages. It is therefore impossible to present the full survey in this paper. This can be done only for Section 1 regarding certification (see Figure 2), which was very short. For the remaining sections, as an alternative approach, we tried to summarize the questions contained in each section, so that the reader can have an overview of the main subject of each section and the various features that DMS may present. The complete survey results will be included in the relevant upcoming IAEA publication.

### 3.1 Section 1: Certifications

As shown in Figure 2, 4 out of 11 DMS developers have Food and Drug Administration (FDA) approval (36%), 7 out of 11 developers (64%) have a CE mark, and 5 out of 11 (45%) have other types of certifications. Most of the DMS developers (8 out of 11; 73%) help the users comply with United States Joint Commission requirements.

### 3.2 Section 2: Data transfer methods, general patient & facility information, correction factors

The questions of this section concern the ways with which the DMS can be connected to the modalities [directly, via Picture Archiving and Communication System (PACS) or other method] to automatically acquire data regarding the patient demographics, facility details, exam type information, exposure parameters, dose metrics, and other data. Also, it is surveyed which of the well‐known methods ‐like Digital Imaging and Communications in Medicine (DICOM) Radiation Dose Structured Report (RDSR), DICOM Patient Radiation Dose Structured Report (PRDSR), DICOM Modality Performed Procedure Step (MPPS), Optical Character Recognition (OCR) of dose report images, DICOM protocol storage, etc., can be employed for this purpose.

The remaining questions of this section concern: a) the procedure with which a record is created after an examination/study is performed in any modality connected to the DMS in any way, and the way that all information that refers to the patient, study, facility and modality, staff involved (operator, requesting and referring physicians), and contrast media information, are collected to populate the respective record fields, and b) the calculation of cumulative dose metrics that are applicable to the selected modality or applicable to all modalities, as, for example, the cumulative DLP values for all the CT examinations performed on the same patient, or the cumulative effective dose (*E*) from all radiological examinations, respectively. Finally, the last question of this section investigates the capabilities of DMS to use correction factors to account for unit conversion from the original reported by the radiological system to the preferred one (e.g., mGy instead of dGy) or to account for calibration errors [e.g., in case that an Air Kerma Area product (KAP) meter is known to overestimate the actual KAP value].

**FIGURE 3:**
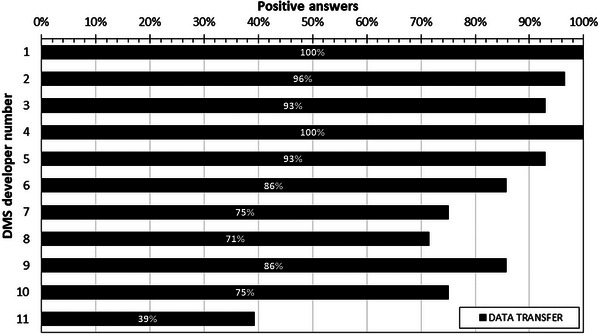
Overview of the percentages of positive answers given in Section 2 (Data Transfer) of the DMS survey.

The results of the analysis of this section are shown in Figure 3. As shown, 7 out of 11 DMS developers (64%) had over 80% positive answers to the questions of Section 2. There was one open‐source system that responded positively to only 39% of the questions in Section 2. The smaller differences in percentages among the rest of the developers are mainly due to the fact that while all developers use the RDSR, and almost all DICOM headers and OCR, other methods like MPPS and PRDSR are not supported by some developers. Furthermore, four developers did not support the transfer of contrast media information, and three did not support the use of correction factors to convert or correct dose metrics’ units and values.

### 3.3 Section 3. Modalities, metrics, and methods supported by the DMS for different modalities

In this section, a detailed series of questions is contained regarding the information collected pertaining to the examination and acquisition protocol, exposure parameters, dose metrics, and additional information about the exam. Each question is analyzed separately for each one of the most common modalities supported by DMS, namely, computed tomography (CT) scanners, interventional and other fluoroscopic fixed and mobile C‐arms systems, radiographic systems (fixed and mobile), mammography systems (2D and digital breast tomosynthesis systems), and finally dental imaging systems [cone‐beam computed tomography (CBCT), panoramic and cephalometric systems]. It must be noted, however, that the list of questions is not exhaustive, as it is well known that the number of parameters recorded, for example, in a plain radiographic DICOM image can be hundreds.

The approach of surveying each modality separately was selected because though all modalities may share many common parameters regarding the patient information, exposure parameters like tube potential (kVp) and tube loading (mAs), etc., there are also many differences regarding other technical parameters. Therefore, there are some identical questions repeated for each different modality, but there are many questions which are applicable only to some or even only one modality. For example, the field of view (image's dimensions) is applicable to fluoroscopy, radiography, and mammography but not to CT where the scan field of view (FOV) and the display FOV are the corresponding information, while the collimation configuration and reconstructed slice thickness, the fluoroscopy time, and the compressed breast thickness (CBT) are applicable only to CT, fluoroscopy, and mammography, respectively.

**FIGURE 4:**
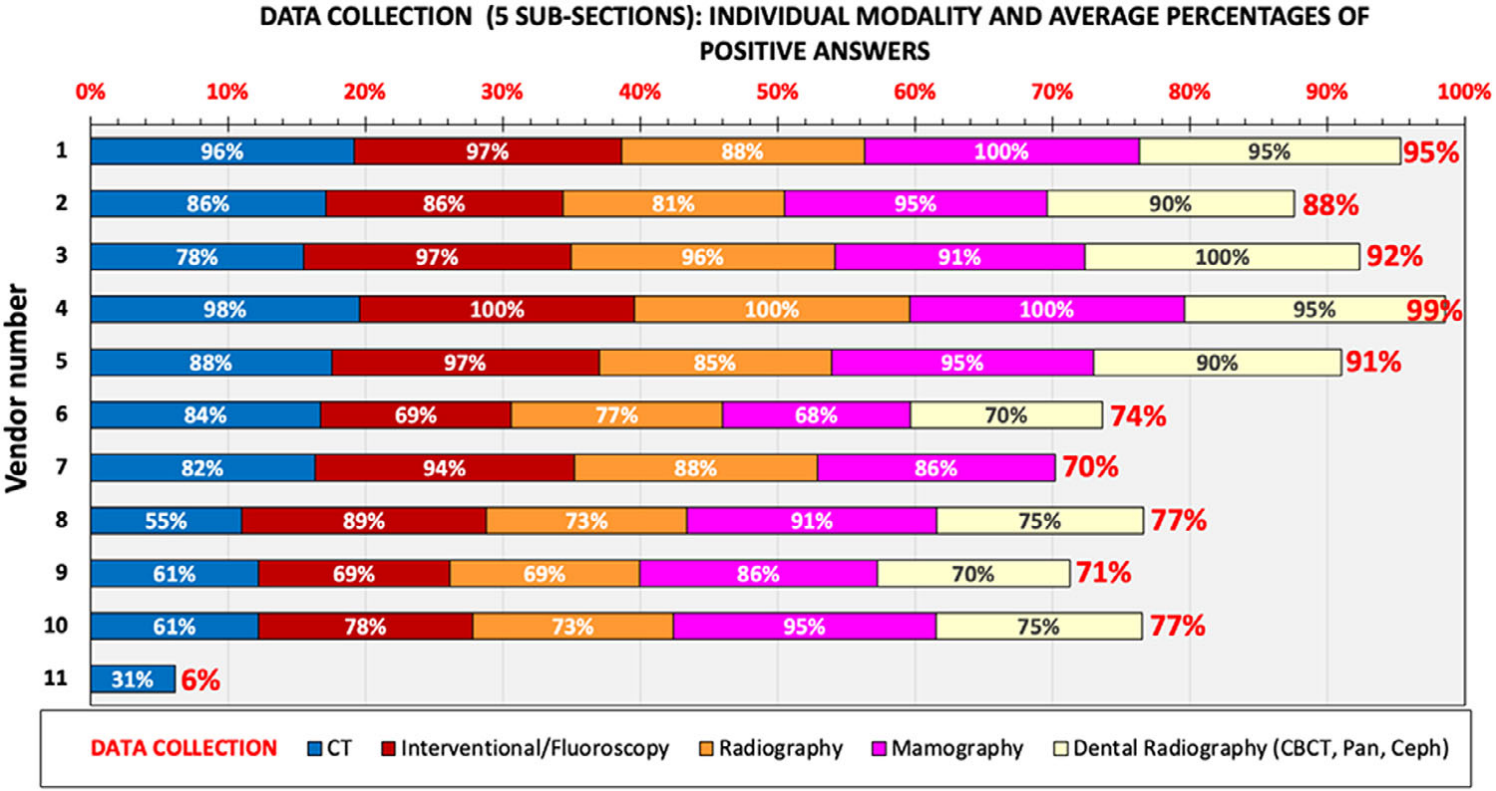
Overview of positive answers given in the five subsections of Section 3 (Data Collection) of the DMS survey. Each bar color corresponds to an individual sub‐question of this section. The total stacked bar length and red data label correspond to the average percentage of positive responses in the entire section.

The questions had to account for the large differences that also exist regarding the dose metrics, both those collected and those calculated by the DMS. For example, for CT scanners, the standard dose metrics transferred to DMS are the volumetric CT dose index (CTDI_vol_) and dose length product (DLP), for fluoroscopy and radiography systems the Kerma or Dose Area Product (KAP or DAP) and the incidence entrance dose to a reference point (per acquisition and cumulative), for mammography the entrance surface air kerma (ESAK without backscatter) and the average glandular dose (AGD), while for dental units (panoramic/cephalometric/CBCT) the typical dose metric transferred is the KAP and in some cases the CTDI also (applicable to CBCT only). The results of the analysis of this section are shown in Figure 4. The average positive response was 76% with none of the DMS developers reporting “Yes” to all questions. Five out of 11 (45%) had over 85% positive responses and one (open‐source DMS) responded positively to only 6% of the questions, which corresponded to 31% of the questions related to CT scanners.

Regarding the dose metrics which are calculated by the DMS using the data transferred from the modalities to the DMS, many questions are pertinent to CT scanners only. This is due to the fact that since CT is one of the most common X‐ray examinations and the main source of radiation dose to the public from the medical use of ionizing radiation. Thus, apart from CTDI_vol_ and DLP, additional quantities have been developed to better quantify the radiation burden to the patient. Examples are the size‐specific dose estimates (SSDE) that correct the CTDI_vol_ values considering the patient size, and the CTDI, DLP, and SSDE weighted per acquisition, to provide a weighted average of the above metrics in CT exams with multiple acquisitions.

Furthermore, though questions regarding the ability of DMS to estimate *E* for adults and children is repeated in most modalities, for CT, this question is broken down into many individual questions, pertinent to the level of accuracy with which *E* is estimated. Questions exist regarding the ability of DMS to estimate *E* using approximate conversion factors for adults or children of specific age groups (which is the standard method used in most modalities) or more sophisticated methods using geometrical or anthropomorphic phantoms, that may or may not match the patient body habitus and the actual planned scan length and automatic tube current modulation (ATCM) data, to provide a more accurate estimate of *E* and organ doses. Another question that is repeated in many modalities, is the presence of indices that refer to the manual or automatic evaluation of image quality.

There are also questions that refer to special parameters which are applicable to CT only and are calculated by the DMS using the scan projection radiographs (SPR) or even the reconstructed CT slices, like the patient diameters (anteroposterior and lateral), the effective and the water equivalent diameter (WED), which are required for the calculation of the SSDE. Furthermore, a few questions are included regarding the extraction of parameters related to the ATCM settings, and manual or automated evaluation of image quality metrics if these are reported by the CT scanner or can be extracted or calculated by the DICOM images. Finally, for CT scanners, a particular question exists regarding the DMS capability to calculate the centering of the patient within the gantry since this may affect the operation of the ATCM and consequently image quality. To appreciate the differentiation of requested details among modalities, for CT scanners, there are 49 questions, for fluoroscopic 36, for radiographic 26, for mammography 21, and for dental CBCT systems 19 questions.

The largest part of the differences observed in the percentages of positive answers between DMS developers regarding the CT scanners sub‐section (Figure 4: blue data columns) were due to functions related to the extraction or calculation of these additional metrics and advanced parameters, which are pertinent to CT scanners only and are not supported by all DMS.

Regarding the interventional fluoroscopic systems sub‐section, the largest part of the differences observed among DMS developers (Figure 4: dark red data columns) are mainly due to DMS functions related to estimation of *E* and the extraction or calculation of information related to the peak skin dose (PSD) and peak skin dose mapping, and the image quality evaluation. PSD is a very crucial quantity since in some complicated examinations the PSD may exceed the threshold for the occurrence of deterministic effects. Though most of these fluoroscopy systems calculate the cumulative air kerma to a reference point as a surrogate of the maximum possible skin dose, there are only a few that may incorporate sophisticated skin dose mapping software (as standard or optional) to calculate the peak skin dose value and give a graphical representation of the skin dose distribution. Some DMS incorporate such algorithms that can do the same thing, provided that the fluoroscopy systems do export to the DMS detailed data (by means of the RDSR or DICOM headers), not only regarding the exposure factors used in each radiation event but also regarding the geometrical conditions that apply in each radiation event, such as the patient orientation, the table and x‐ray tube relative positions and angles, FOV, etc.

Regarding the radiographic systems sub‐section, the largest part of the differences observed among DMS developers (Figure 4: orange data columns) are mainly due to DMS functions related to estimation of *E* and image quality evaluation. For mammography systems sub‐section, the differences observed among developers (Figure 4: pink data columns) are due to the fact that there are many parameters which for some reason are not collected from some DMS. There were also three developers that do not collect data on magnification, automatic exposure control (AEC) mode used, and image quality evaluation. It was also surprising to see that one DMS developer does not collect ESAK data. Regarding the dental systems sub‐section, the largest part of the differences observed among developers (Figure 4: light yellow data columns) are mainly due to DMS functions related to estimation of *E*, image quality evaluation, and information regarding additional filter used and AEC (if applicable). One developer that supported all the previously discussed modalities did not support dental systems (no positive answers to any of the questions).

It should be noted that Section 3 had two additional questions (not included in the graphs), which referred to whether DMS supports occupational dose tracking (2 out of 11 DMS developers), and other modalities like Magnetic Resonance Imaging (MRI), ultrasound, etc. (6 out of 11 DMS developers). Regarding occupational dose tracking, the ICRP recommends that “Occupational exposure in interventional procedures is closely related to patient exposure and, therefore, occupational protection should be managed in an integrated approach with patient protection.”^19^ Therefore, to record the occupational dose in a DMS together with the patient dose indicators can be of great help in the process of optimization of interventional procedures.

### 3.4 Section 4. Statistical analysis capabilities provided by the DMS

The questions of this section refer to the features that DMS may have regarding the statistical analysis of the collected or calculated data.

The questions included in the first sub‐section are pertinent to the ways that the results of statistical analysis can be presented in preset or customizable dashboards. The most common requirement relevant to regulatory compliance for the DMS, is to be able to present a column graph with the median values of a dose metric (e.g., DLP), in a specific exam (e.g., CT routine chest), across all CT scanners of an organization, of a hospital or of a specific manufacturer, in comparison with the diagnostic reference level (DRL) value that has been established for this examination (international, national, regional, local, or institutional DRLs). Filters to adjust the time period from which these data are extracted commonly exist, as well as other filters that classify data depending on patient body habitus using indices like weight or body mass index (BMI), WED, or age. The presence of DMS features that support the calculation of the 75‐percentile of such distributions is also investigated, for setting local or institutional DRL (in case that international or national DRL do not exist). Detailed questions also exist regarding standard and alternative ways which may be used by DMS to present the results of statistical analysis using preset or customizable graphs and tables, and other special functions like reject and repeat analysis in digital radiography.

The questions included in the second sub‐section are pertinent to certain queries that DMS can perform, in order to investigate some particulars of the examinations performed in any modality using one or multiple criteria regarding collected data or calculated parameters. These can be simple queries like “report the number of Chest CT studies that were performed during last year per facility,” or more complex like “report the number of chest CT exams with SSDE values in the range 200−400 that were performed last year per facility,” or “report the number of radiographs performed last week per facility, where a kVp value within the range 50−60 was used.” These questions were used to investigate whether the DMS can be used to perform multiple criteria analysis of the stored data, that can be used for numerous purposes, which can be as simple as the investigation of adherence to certain guidelines related to optimum techniques, more complex like identification of wrong practices that could, for example, be observed mostly during afternoon or night shifts, as well as, for other administrative or research purposes.

Finally, the third sub‐section includes questions regarding the ways in which results of the aforementioned DRL compliance checks, and of other statistical analysis tasks and specialized investigations can be reported. Also, questions are included regarding the capability of the DMS to export data to be further analyzed using other software (e.g., Microsoft Excel), and the capability to customize the DMS data filtering, so that part of data is exported only, depending on the information detail required for a specific task.

**FIGURE 5:**
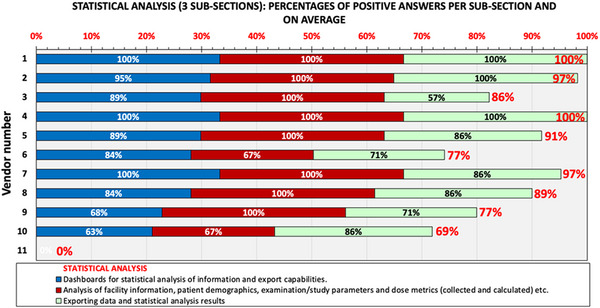
Overview of positive answers given in the three sub‐sections of Section 4 (Statistical Analysis) of the DMS survey. Each bar color corresponds to an individual sub‐question of this section. The total stacked bar length and red data label correspond to the average percentage of positive responses in the entire section.

The results of the analysis of this section are shown in Figure 5, where the percentages of positive answers in each sub‐section are shown with different column color, while the average is given as stacked column (red label values).

### 3.5 Section 5. Customization of DMS (alerts, master protocols, DRL libraries, user rights)

The first sub‐section of this section includes many questions used to understand how the DMS are customized to produce alerts when certain values of individual or cumulative parameters of collected data or calculated quantities that may refer to a single acquisition or complete study, are below, above, or not within certain reference values. Various questions are used to understand how these alerts are manifested (e.g., using graphic symbols or colors) to inform the user that some limits have been exceeded and action has to be taken, and what kind of action has to be taken to resolve such alerts.

The second sub‐section of this section includes questions designed to examine the methods that DMS use to solve a very common problem, which is the usage of different names across the various facilities within the same organization to describe practically the same examination or acquisition protocol. A solution to this problem in most DMS is the creation of categories that sometimes are called “master protocols,” under which all different named protocols used for the same examination/acquisition are categorized, so that the DMS will group them all together as being the same examination/acquisition. Questions are designed to understand the different ways DMS use to set different levels of alerts for each master protocol, for example, code orange (level 1 alert) or code red (level 2 alert), for dose metrics or other parameters. Furthermore, the answers to the questions provide information on how these limits can be stratified depending on body habitus, patient age or clinical indications, or are created manually or automatically using data from established international, national, regional, or any other DRL values and various pre‐existing or customizable DRL libraries. Furthermore, questions that relate to the ways that an unknown examination protocol is managed and assigned to a Master protocol are also included. It must be noted that regarding to examinations of the same anatomic area but for different clinical indications, since the image quality level and consequently the dose level required may differ much, an effective way to account for this is the use of different master protocols for different clinical indications. Alternatively, a DICOM tag information related to clinical indication can be used to stratify the reference values for the alerts.

The third sub‐section is comprised of questions that refer to preset DRL libraries and customizable DRL libraries as well as on the ways that these can be uploaded, updated, or created from scratch. There are also questions that refer to safeguards that exist to inform and/or alert the system administrator regarding intentional or unintentional modification of DRL or reference values/limits libraries, or for contradicting and/or overlapping ranges in DRL stratification settings. The fourth sub‐section includes questions related to user rights such as, for example, who and how he/she is allowed to manage and/or modify DRL libraries, reference values and limits, resolve alerts, etc.

**FIGURE 6:**
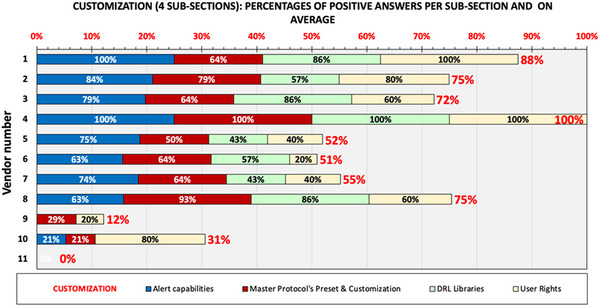
Overview of positive answers given in the four sub‐sections of Section 5 (Customization) of the DMS survey. Each bar color corresponds to an individual sub‐question of this section. The total stacked bar length and red data label correspond to the average percentage of positive responses in the entire section.

The results of the analysis are shown in Figure 6, where the percentages of positive answers in each sub‐section are shown with different column color, while the average is given as stacked column (red label values). Section 5 appears to have the largest differences between DMS developers ranging from 0% to 100% with an average of 59%. Only three DMS developers managed to respond positively to more than 80% of the questions, whereas there were three of them with less than 40% (DMS developers 9, 10, and 11). It is thus obvious that customization possibilities vary greatly among developers.

### 3.6 Section 6. Implementation process (IT, installation plan, IT requirements, service and update schedule)

**FIGURE 7:**
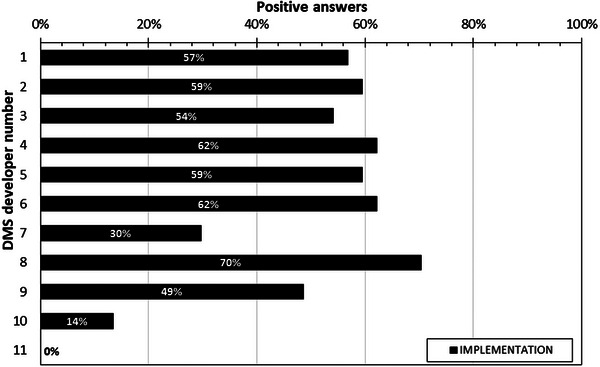
Overview of the percentages of positive answers given in Section 6 (Implementation) of the DMS survey.

Section 6 is the most sophisticated part of the survey. It has four sub‐sections and includes many complex technical questions to understand what are the DMS requirements regarding Information Technology (IT) support within the organization, what are the minimum and desired hardware requirements, what is the plan and what are the steps required to complete the installation regarding connections with PACS, the modalities, initial customization, and expected time duration for the process, what are the training requirements and time schedule for the DMS administrator and the simple users. Furthermore, what are the safeguards and precautions against loss of data due to hard disk failure or connection problems with the cloud or the individual modalities, and what are the abilities of the DMS to transfer data to various registries such as the American college of Radiology (ACR) Dose Index Registry, or other services related to electronic patient records. The remaining questions refer to guarantee, maintenance and service information, software updates, and to the procedure that must be followed to connect to the DMS additional modalities. The results of the analysis of this section are shown in Figure 7. DMS developers did not provide answers to all questions of the Section. The average positive response was 47% with a range of 0−70%.

**FIGURE 8:**
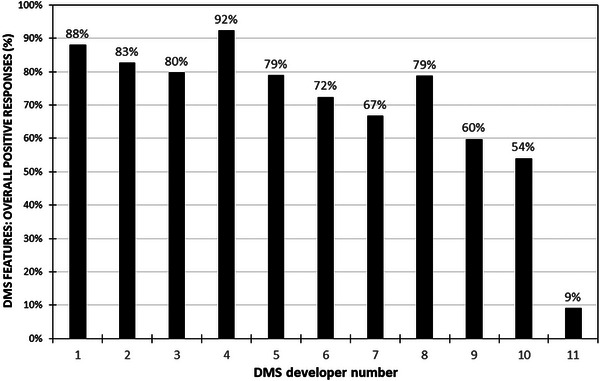
Overview of overall positive responses of the DMS survey.

Finally, Figure 8 is a graphic summary of the overall affirmative feedback recorded in this survey. It is evident that for the majority of DMS developers (7 out of the 11), a substantial positive response rate exceeding 70% to the posed inquiries was recorded, indicating that the majority of DMS developers currently offer a broad range of functionalities.

## 4 DISCUSSION

In this survey, a detailed overview of the majority of the existing DMS was carried out, regarding their features and capabilities. The survey questions were designed based on our current understanding of the functionalities and capabilities of various DMS, in order to assess their suitability for end‐users. The questions considered factors such as software features in general and per modality, clinical requirements, skill levels, sustainability considerations, and the availability of technical support. Furthermore, the survey aimed to identify potential areas for improvement of these systems’ capabilities. To the best of our knowledge, this research represents the inaugural study of its kind, as evidenced by the lack of similar literature in the field. Given the absence of comparative literature, the study does not only establish a crucial benchmark but also highlights numerous potential directions for future research. One example is the way that different DMS developers deal with the problem of lack of standardization on examination protocol nomenclature, which is typical in CT but also applies to interventional procedures. This may concern simple CT exams of the same anatomical area but different clinical indications, and multi‐phase CT studies that may contain a different number of scans depending on the clinical findings (e.g., delayed acquisitions). A second example is the method or methods that DMS developers use to estimate parameters like *E* or peak skin dose, the documentation of the method(s) offered and the calculated uncertainty of each method.

The main results of this survey are summarized in Figures 3, 4, 5, 6, 7, which exhibit the differences that exist between different DMS developers regarding DMS capabilities. However, it must be emphasized that the percentages appearing in Figures 3, 4, 5, 6, 7 are simply a measure of positive answers and should not be considered as an absolute index for benchmarking the DMS regarding their capabilities. Though a greater number of positive answers indicate a more complete system based on our questionnaire, no extra weight is given in some important capabilities that some systems offer, and some systems do not, as, for example, calculation of parameters like WED and peak skin dose, or automatic image quality evaluation algorithms. It is understandable that some may think that DMS which provide sophisticated features, such as organ dose calculations in CT exams and peak skin dose in interventional fluoroscopy procedures, should be considered state‐of‐the‐art and should be clearly considered superior to other DMS which do not have such features. However, in the type of analysis used, having these sophisticated features is just one more positive answer, which has the same weight as those questions that refer to common features. Hence, this is an important reason why the survey's outcomes presented in Figures 3, 4, 5, 6, 7 should not be strictly considered as benchmarking.

Furthermore, regarding the sophisticated features, some may not be applicable for some potential users, such as, for example, the peak skin dose calculation, which will work only if the fluoroscopy systems export the required information. Also, the accuracy and uncertainties of these calculations cannot be known if the DMS does not provide relevant documentation or/and relevant studies from the international literature that have validated the accuracy of the methods used by the DMS. This holds true as well for elaborate organ dose calculations in CT scanners. Moreover, at this point, the potential extra cost associated with the incorporation of such an option is unknown. The degree of accuracy of a sophisticated feature and its associated cost needs to be thoroughly examined prior to any purchase decision. A prospective user must consider the substantial financial investment involved, and evaluate the added value derived from the extra cost. As far as *E* is concerned, despite the fact the accuracy and use of *E* is not within the scope of this study, it should be mentioned that ICRP, in its document no. 147, recommends against the use of *E* for patient dose tracking in medical exposures.^20^ Therefore, the effective doses provided by many DMS should be used following the ICRP recommendations.

Section 6 (Implementation) was the least completed section of the survey, probably because it was too detailed and/or because hardware specifications change too rapidly following software updates. Another possible reason is that the installation plan may vary considerably depending on the existing IT infrastructure within the organization and the user's selection with respect to whether data will reside (local server or cloud solutions), making difficult for the DMS developers to provide exact answers.

It must also be noted that although a DMS developer can offer a specific functionality, this may not be granted for all x‐ray systems connected. For example, even though a DMS developer can offer recording of SSDE in CT, some CT manufacturers do not provide SSDE metrics at all, and others may provide it but place it not in the RDSR but in a private tag that is not accessible to the user. Some data elements that are relevant or necessary for a DMS to calculate certain parameters, are identified as “type 3” or requirement type “user option” data elements in the DICOM standard.^21^, ^22^ This practically means that x‐ray system manufacturers are not obliged to include the particular DICOM information of the relevant studies. In other words, it is not necessary to include some of the parameters of interest for dose management in the studies’ information to be DICOM compliant. Therefore, to ensure full functionality of DMS, it is essential that x‐ray system manufacturers provide all the information recommended by the DICOM standard in their RDSR reports.

It is worth noting a significant limitation of this survey is the human resource aspect which was not explored in detail. The successful integration of a DMS is not merely a technological endeavor; it is equally contingent on the capabilities and adaptability of the staff who will be utilizing the system. Unfortunately, our survey did not investigate the potential human resource needs related to the adoption and operation of a DMS, such as the necessity for technical skills or additional training. Moving forward, future research should address this gap by considering the human resources component in detail. This will provide a more holistic view of the considerations involved in selecting a DMS solution, contributing to more informed decision‐making within healthcare facilities.

## 5 CONCLUSION

The results of this survey clearly exhibit that large differences exist between the various DMS developers currently available, as shown by the answers of those who participated in this survey. Consequently, healthcare institutions contemplating the acquisition of a DMS solution should comprehensively explore the available DMS solutions, regarding the features and functionalities that they offer, to make sure that they align with their specific needs. Subsequently, they must identify which of those advanced features, which may be optional and come at an extra cost, are either essential or desirable for their specific organization, considering the available budget. Finally, it should be confirmed whether the existing infrastructure and information technology personnel available are compatible with the proposed DMS installation, operation, and service support requirements. This approach could potentially optimize expenditure, ensuring a balance between operational efficiency and budgetary constraints.

## AUTHOR CONTRIBUTIONS

All authors substantially contributed to the conception or design of the research and analyzed/interpreted results. Also, all authors contributed to drafting the manuscript or revising it critically for important intellectual content.

## CONFLICT OF INTEREST STATEMENT

The authors have no conflict of interest to state.
